# Structural Properties and Hydrolysability of *Paulownia elongate*: The Effects of Pretreatment Methods Based on Acetic Acid and Its Combination with Sodium Sulfite or Sodium Sulfite

**DOI:** 10.3390/ijms23105775

**Published:** 2022-05-21

**Authors:** Hanxing Wang, Ni Chen, Feifan Xie, Erkki Verkasalo, Jie Chu

**Affiliations:** 1 College of Forestry, Northwest A&F University, Yangling, Xianyang 712100, China; whxing1121@163.com (H.W.); 119@nwafu.edu.cn (N.C.); ff66997757@163.com (F.X.); 2 Natural Resources Institute Finland (Luke), Production Systems, Yliopistokatu 6, 80100 Joensuu, Finland; erkki.verkasalo@luke.fi

**Keywords:** *Paulownia*, enzymatic hydrolysis, combined pretreatment, structural properties, yield

## Abstract

The effects of CH_3_COOH and Na_2_SO_3_ pretreatment on the structural properties and hydrolyzability of fast-growing *Paulownia elongate* were investigated. Acetic acid increased cellulose’s crystallinity and hydrolyzability when combined with alkaline sodium sulfite and sodium hydroxide. The cellulose content increased by 21%, the lignin content decreased by 6%, and the product showed better enzymatic digestibility. With a cellulase dose of 30 FPU/g DM, after 72 h hydrolysis, the hydrolysis yields of glucose and xylose were 78% and 83%, respectively, which were 51% and 69% higher than those of untreated materials. When the enzyme dosage was 20 FPU/g DM, after 72 h hydrolysis, the hydrolysis yields of glucose and xylose were 74% and 79%, respectively. The high hydrolyzability, low enzyme loading, and high hydrolysis yield demonstrate the potential of the proposed system for producing platform sugars from fast-growing *Paulownia elongate*.

## 1. Introduction

Biomass energy is the fourth-largest energy source in the world [1]. With the development of the bioenergy industry, more biomass materials, such as energy grass, energy crops, crop straw, low-value wood, wood waste, etc., have gained much attention in recent years. They are used to produce a variety of bioenergy materials, but increasing their use requires more evaluation for green renewable utilization. The genus *Paulownia* comprises nine fast-growing, relatively short-term multipurpose species native to China and Southeast Asia [2]. They are important in China due to their many uses [3,4,5] and ability to grow in poor soils where they do not compete with crops for nutrients [6,7,8]. China contains 27% of the global Paulownia resources, with a total plantation area of 9.68 million hectares [2,9]. Based on the before-mentioned benefits, *Paulownia* species have been introduced in many countries, including Spain, Italy, Austria, Turkey, Israel, India, the United States, Canada, Mexico, and Brazil [10]. Paulownia is not comprehensively utilized. In China, the utilization rate of timber is less than 25%, and it is mainly used for wood veneer processing, wood furniture, and medicinal applications [2,6]. Paulownia processing provides about 30 million cubic meters of residues every year, about 90% of which are not used efficiently [2,11]. In China, these waste wood resources can supply 90 million liters of E3 gasoline through biodegradation, saccharification, or fermentation while producing wood fiber and liquid fuels [12].

According to previous research, the wood material extracted from Paulownia is a rich organic resource that contains 50–60% cellulose, 30–40% hemicelluloses, 10–15% lignin, and small amounts of ash and extractives (dry mass basis) [13,14,15]. Compared with wood-based materials from most other species, the lignin content is relatively low in Paulownia. Lignin can be used as an abandoned fuel with other residues that cannot be converted into ethanol. Paulownia processing residues also contain less ash and soluble materials [16]. More importantly, the cellulose and hemicelluloses contents are relatively high, providing an advantage for subsequent saccharification, hydrolysis, and fermentation of wood fiber materials [17]. *Paulownia* is an interesting commercial species for bioenergy and biorefining that can simultaneously fix carbon and reduce greenhouse gas emissions; thus, Paulownia has become an important raw material for ethanol production. Accordingly, Paulownia biomass materials were studied in Spain for acid pretreatment cracking degradation, achieving about a 95% ethanol conversion rate [18]. The production technology of cellulose ethanol from abandoned Paulownia timber was officially put into production in Osaka, Japan in 2016 [18]. The bioenergy conversion of Paulownia wastes would be an effective process for their utilization in China instead of burning them.

However, there are few studies available on ethanol production from Paulownia processing residues in China. Since the 1950s, China has developed many key technologies for the hydrolysis and saccharification of pretreated raw materials, such as dilute acid atmospheric pretreatment, dilute acid pressure pretreatment, concentrated acid-liquid ratio hydrolysis, cellulase hydrolysis, etc., which produce 180 L of alcohol per ton of material and 38–40% of hydrolyzed monosaccharides [19]. In 2013, the project team of the South China University of Technology first used microwaves and sodium hydroxide to treat *Paulownia tomentosa* with a hydrolysis yield of 65–70% xylan [20]. The technology for preparing ethanol fuel via the hydrolysis and saccharification of wood cellulose waste in China is still in its infancy. Key degradation technologies such as cellulase fermentable sugar preparation and pentose-hexose synchronous ethanol fermentation of wood biomass raw materials are not mature enough. The hydrolysis technology is still quite different from that of other countries, and the economics are poor [21]. Due to the diverse raw materials used to prepare ethanol, various production processes are used, and a general method that could break the rigid structure of biomass into a form that is more conducive to enzymatic hydrolysis is usually employed.

In recent years, combined pretreatment technologies have become the most widely used and environmentally friendly pretreatment technologies for lignocellulosic raw materials. Many scholars have used two solvents to pretreat bamboo and for the enzymatic hydrolysis of spruce [22,23]. Using different biomass materials, relatively low hydrolysis yields (<45%) have been achieved even with a high substrate addition (15%) [24,25]. A feasibility study [26] showed that collaborative pretreatment has the following effects: (1) it destroys the three-dimensional structure of lignocellulosic biomass and reduces the polymerization degree and crystallinity of cellulose [27]; (2) it increases the surface area and porosity of a material [28]; and (3) hydrolysis produces a pretreated solid with high digestion that promotes the yield of sugars [29]. At the same time, the specific surface area of lignocellulosic materials is multiplied by synergistic pretreatment, which means that the accessibility of various cellulose, hemicellulose, and lignin molecules in the fiber structure is improved. The treatment efficiency of hydrolysis, enzymolysis, or organic solvents is improved [30]. That is, the synergistic process destroys the cell walls and the fibrous web structure and promotes the accessibility of organic solvents and the transformation of internal substances during extraction [31]; however, whether the pretreatment of Paulownia has the above characteristics after enzymatic hydrolysis requires further research.

The objective of this study was to investigate the effects of three pretreatment systems for fast-growing *Paulownia elongate* from China on its structural properties and hydrolyzability. A comparison was made to non-treated wood. Acetic acid was combined with sodium sulfite or alkaline sodium sulfite. The hydrolysis saccharification rate was analyzed using different cellulase enzyme dosages and hydrolysis times. The main chemical components were determined using the National Renewable Energy Laboratory (NREL) method. Structural characterization was performed using scanning electron microscopy (SEM), Fourier-transform infrared (FT-IR) spectroscopy, thermogravimetric analysis (TGA), and X-ray photoelectron spectroscopy (XPS). The study investigated the recovery of sugar extraction for further fermentation via bioethanol production and the most efficient process to recover all available fermentable sugar. Accordingly, this study provides a basis for further improving the utilization of Paulownia resources by hydrolytic saccharification, which is an environmentally friendly and sustainable method for using lignocellulosic resources.

## 2. Materials and Methods

### 2.1. Materials

The raw material used in this study was the stem wood residues of processed *Paulownia elongate* supplied by the National Forestry Bureau, Paulownia Research and Development Center (Zhengzhou, China). The material was cut into shavings, dried, and then pulverized into fine granules by a pulverizer. Thereafter, the material was milled and sieved through an 80-mesh screen scale and stored in a plastic bag at room temperature until further experiments. The moisture content (MC) of the pulverized raw material was kept below MC 10% (dry mass basis).

### 2.2. Pretreatment

Three pretreatment methods were applied for the three milled samples, with a control without pretreatment. Samples were analyzed for each method and reference.

All pretreated samples were first pretreated with 5 wt% acetic acid at 170 °C for 0.5 h (after soaking the dry matter in acetic acid solution for 1 h at ambient temperature), using a high solid-to-liquid ratio of 1:10 (weight/volume). After the treatment of acetic acid, the filter residue was washed with distilled water to neutral, dried, and bagged. An oil bath was used for heating during the experiments, and the reaction vessel was a stainless-steel reaction kettle.

Of the acetic acid samples, one part was further pretreated with 4 wt% Na_2_SO_3_, and others were pretreated with 4 wt% Na_2_SO_3_ with 1 wt% NaOH (these were called the alkaline sodium sulfite treated samples). Both treatments were applied at 121 °C for 1 h with a solid-to-liquid ratio of 1:10. The supernatants and solids were separated from each sample by centrifugation (5000× *g*, 10 min). The acetic acid in the pretreatment supernatant was counted to the recovery.

All pretreatment residues were washed with distilled water until the pH was neutral. After that, the solids were air-dried and stored at −19 °C for later experiments and structural characterization.

### 2.3. Enzymatic Hydrolysis

The content of non-structural monosaccharides in pretreated and non-treated samples was studied using enzymatic hydrolysis to determine the yields of glucose and xylose. The glucose yield was used to evaluate the hydrolysis performance after different treatments.

Hydrolysis was employed to prepare cellulase and was performed in tubes with a working volume of 1.5 mL in 50 mM sodium citrate buffer (pH 5.0) at 50 °C. Hydrolysis was conducted in a shaking incubator at 200 rpm. The dry matter (DM) content of the substrate was 2%. NaN_3_ (0.02%) was added to the hydrolysis broth to prevent bacterial infection. Cellulase preparation (CEL) used Cellic^®^CTec2(Novo Nordisk A/S, Denmark). To investigate the effects of enzyme dosage on enzymatic hydrolysis, the cellulase doses were 10, 20, and 30 FPU/g DM, and the hydrolysis time was 72 h.

To study the effect of hydrolysis time, enzymatic hydrolysis times of 48 h and 72 h with an enzyme dosage of 20 FPU/g DM were used. Boiling for 10 min was used to stop the enzymatic hydrolysis. After cooling, the samples were centrifuged at 10,000× *g* for 10 min, and the glucose and xylose contents of the supernatants were analyzed using HPLC. Two replicates were performed in all hydrolysis experiments, and their average values were used as the final experimental data for each sample.

### 2.4. Analytical Methods

The chemical composition of the samples was determined by the standard method of NREL [32,33]. The contents of cellulose, hemicelluloses, acid-insoluble lignin, acid-soluble lignin, extractives, and ash in the non-treated and pretreated samples were determined based on the NREL analytical procedure. All component analysis experiments were carried out in three sets of parallel experiments, and the final data were expressed as the average values.

The glucose and xylose contents were determined using a standard high-performance liquid chromatography system (Hitachi L-2000, Hitachi Ltd., Tokyo, Japan). The system was equipped with a refractive index detector and an autosampler. An ion-moderated partition chromatography column (Aminex column HPX-87H, Bio-Rad, Hercules, CA, USA) with a Cation H micro-guard cartridge was used. The column was maintained at 45 °C with 5 mM H_2_SO_4_ as the eluent at a flow rate of 0.5 mL per minute. Before injection, the samples were filtered through 0.22 μm MicroPES filters, and an injection volume of 20 μL was used. The peaks were detected by refractive index and identified and quantified by comparison to the retention times of D-glucose and D-xylose standards.

The glucose and xylose yields from the hydrolysis of non-pretreated and pretreated samples were calculated using the following equations:

(1) $$\mathrm{Glucose}\;\mathrm{yield}\;\left(\%\right)=\frac{\mathrm{Glucose}\;\mathrm{released}\;*\;0.9}{\mathrm{Theoretical}\;\mathrm{amount}\;\mathrm{of}\;\mathrm{cellulose}\;\mathrm{in}\;\mathrm{substrates}}\times 100$$

(2) $$\mathrm{Xylose}\;\mathrm{yield}\;\left(\%\right)=\frac{\mathrm{xylose}\;\mathrm{released}\;*\;0.88}{\mathrm{Theoretical}\;\mathrm{amount}\;\mathrm{of}\;\mathrm{xylan}\;\mathrm{in}\;\mathrm{substrates}}\times 100$$

### 2.5. Characterization of Solid Residues

Scanning electron microscopy (SEM) was used to visualize the different morphologies of non-treated and pretreated samples. Prior to imaging, the dried samples were coated with gold to render them conductive. Imaging was then performed using an S3400 SEM with an accelerating voltage of 10 kV. All observations were made at 3000× magnification.

For Fourier-transform infrared (FT-IR) spectra, a small amount of each sample was pressed into a disc with KBr and then analyzed on an FT-IR spectrophotometer (Nicolet FTIR IS10, Thermo Fisher Scientific, Waltham, MA, USA). The background spectrum of a diamond window without a sample was subtracted from each sample spectrum. In total, 64 scans were collected for each measurement over the wavelength range of 4000–400 cm^−1^.

For thermogravimetric analysis (TGA), a Q50 thermogravimetric differential thermal analyzer (TGA/DSC) was employed, using nitrogen gas as the shield gas (Shimadzu, Tokyo, Japan). The shield gas flow rate was set to 20 mL/min; the heating rate was 10 °C/min from 25 °C to 600 °C.

For X-ray photoelectron spectroscopy (XPS), the binding energy of the neutral carbon (C–C and C–H bonds) peak was set to 284.8 eV [34,35]. The integrated intensities of the XPS peaks were used to calculate the atomic percentages of various elements. The surface coverages of lignin (*S*_lig_) and carbohydrates (*S*_carb_) were calculated based on the average O/C values using the following equation:

(3) $$S_{\mathrm{lig}}=(O/C_{\mathrm{pretreated}}-O/C_{\mathrm{carbohydrate}})/(O/C_{\mathrm{lignin}}-O/C_{\mathrm{carbohydrate}})\;*\;100$$

### 2.6. Statistical Analysis

Three replicate experiments were performed for each sample. The data were analyzed using one-way analysis of variance (ANOVA) and Tukey’s test at a significance level of *p* < 0.05 with SPSS 19 software (SPSS Inc., Chicago, IL, USA).

## 3. Results and Discussion

### 3.1. Chemical Composition of Paulownia elongate Samples

The main results of the chemical composition of the *Paulownia elongate* samples are shown in Table 1. The contents of cellulose, hemicelluloses, and lignin in the non-treated samples were 47%, 19%, and 32%, respectively. For comparison, Ates et al. found that the holocellulose content was 67% and the Klason lignin content was 23% for *Paulownia elongate* [36]. Garcia et al. concluded in their literature review that the holocellulose content of Paulownia was higher than that of *Paulownia fortunei* and its hybrids with other Paulownia species but slightly lower than of *Paulownia tomentosa* [37].

After pretreatment with acetic acid, the contents of the three major chemicals in *Paulownia elongate* samples were 50%, 11%, and 38%, respectively. The hemicellulose content was 8% lower, possibly because acetic acid has a strong degradation and dissolution effect on xylan in hemicelluloses. During acetic acid pretreatment, hydronium ions degrade hemicelluloses into xylooligosaccharides in the supernatant, which exposes more cellulose to the pretreatment residue. This allows the dissolved xylooligosaccharides to be collected, which improves the comprehensive utilization of biomass materials; however, acetic acid pretreatment has a very limited ability to remove lignin, so the complex crosslinked structure between the three chemical groups must be further destroyed to improve the enzymatic hydrolysis efficiency.

The contents of the three major chemical groups after the combined pretreatment using acetic acid and sodium sulfite were 58%, 9%, and 34%, respectively. Accordingly, the pretreatment removed some lignin, accompanied by the degradation of some xylan, which increased the cellulose content. The complex structure between cellulose, hemicelluloses, and lignin was further damaged.

The contents of the three major chemical groups after the synergistic pretreatment of acetic acid, alkaline sodium sulfite, and sodium hydroxide were 67%, 5%, and 26%, respectively. Compared with the combined pretreatment with sodium sulfite, this pretreatment method had a relatively better delignification effect and degraded some xylan, which greatly increased the cellulose content. The results indicated that the addition of sodium hydroxide facilitates the removal of lignin and extractives.

### 3.2. SEM Analysis

**Figure 1:**
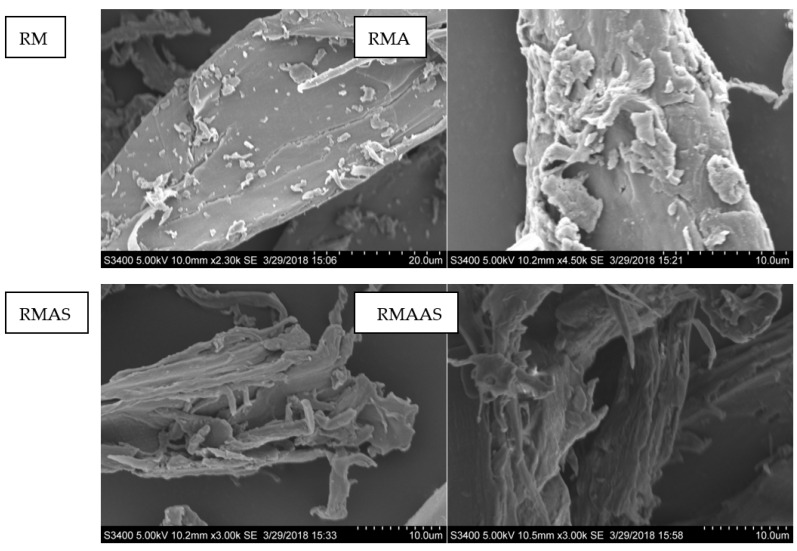
SEM analysis of selected representative samples from different pretreatment conditions. RM: Raw material, no pretreatment. RMA: Raw material + 5% acetic acid. RMAS: Raw material + 5% acetic acid + 4% Na_2_SO_3_. RMAAS: Raw material + 5% acetic acid + 4% Na_2_SO_3_ + 1% NaOH.

The SEM observations indicated that the original *Paulownia elongate* surface was regular with a distinct rigid surface structure and a relatively well-oriented ultrastructure (Figure 1). This structure can hinder the interactions between cellulose and cellulase. Pretreatment with acetic acid formed a slightly disordered morphology, possibly due to the removal of some xylan and extracts. In addition, compared with acetic acid pretreatment, pretreatment with sodium sulfite caused more damage to the rigid structure of the original Paulownia, and an obvious microfibrous structure was observed. The synergistic pretreatment with alkaline sodium sulfite caused the greatest damage to the rigid structure of Paulownia: more microfibers were created, and more cellulose was exposed. This may be because alkaline sodium sulfite accelerated the removal of lignin and extractives. In addition, all pretreatments improved the hydrophilicity and surface roughness of Paulownia. Previous results indicated that an increased roughness promotes enzymatic hydrolysis [38].

### 3.3. FT-IR Analysis

**Figure 2:**
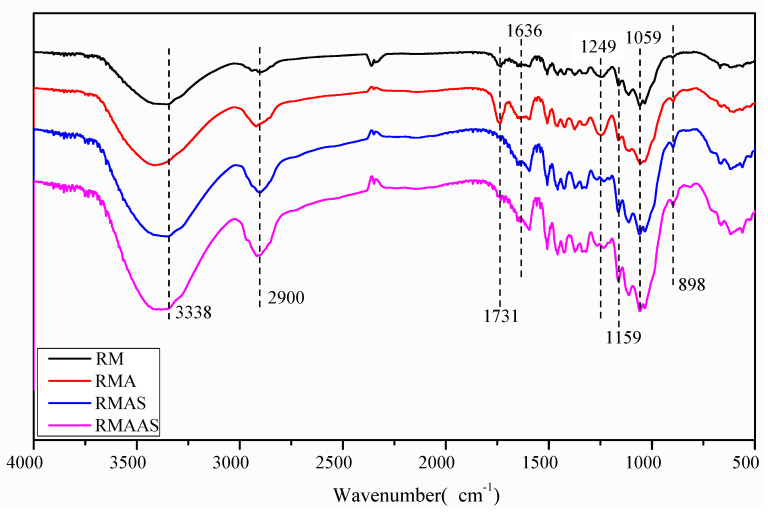
FT-IR analysis of samples under different pretreatment conditions.

The FT-IR spectra of the *Paulownia elongate* samples are shown in Figure 2. The absorption peak at 1731 cm^−1^ represents the ester bond of the hemicellulose acetyl and aldolate groups or the ferulic acid in lignin to the carboxyl group in coumaric acid [39]. The intensity of the absorption peak at 1731 cm^−1^ in the spectrum of pretreated Paulownia was significantly reduced, and the order of peak intensity was non-treated Paulownia > acetic acid pretreated Paulownia > Na_2_SO_3_ pretreated Paulownia > alkaline sulfite pretreated Paulownia. This indicated that the lignin carbonyl was degraded by the pretreatment and caused lignin dissolution. The decreased absorption indicated greater damage to the lignin structure, which made cellulose more accessible to cellulase [40]. Different degrees of structural fragmentation reduced the recalcitrant properties of Paulownia samples and enhanced enzymatic hydrolysis. Alkaline sodium sulfite pretreatment showed a greater ability to destroy these structures. The characteristic peak of lignin was located at 1249 cm^−1^ (C-O guaiac ring), and the peak strength of lignin in non-treated samples was significantly higher than that in pretreated samples. This may be due to a decreased lignin content. Compared with the non-treated samples, the pretreated samples had an increased peak intensity (characteristic peak of β-D glycosidic bond) at 898 cm^−1^ and 1159 cm^−1^, and the reverse order of peak intensity was non-treated Paulownia < acetic acid pretreated Paulownia < Na_2_SO_3_ pretreated Paulownia < alkaline sulfite pretreated Paulownia. This may be due to a higher content of polysaccharides (cellulose and xylan) after pretreatment [41].

### 3.4. TGA Analysis

**Figure 3:**
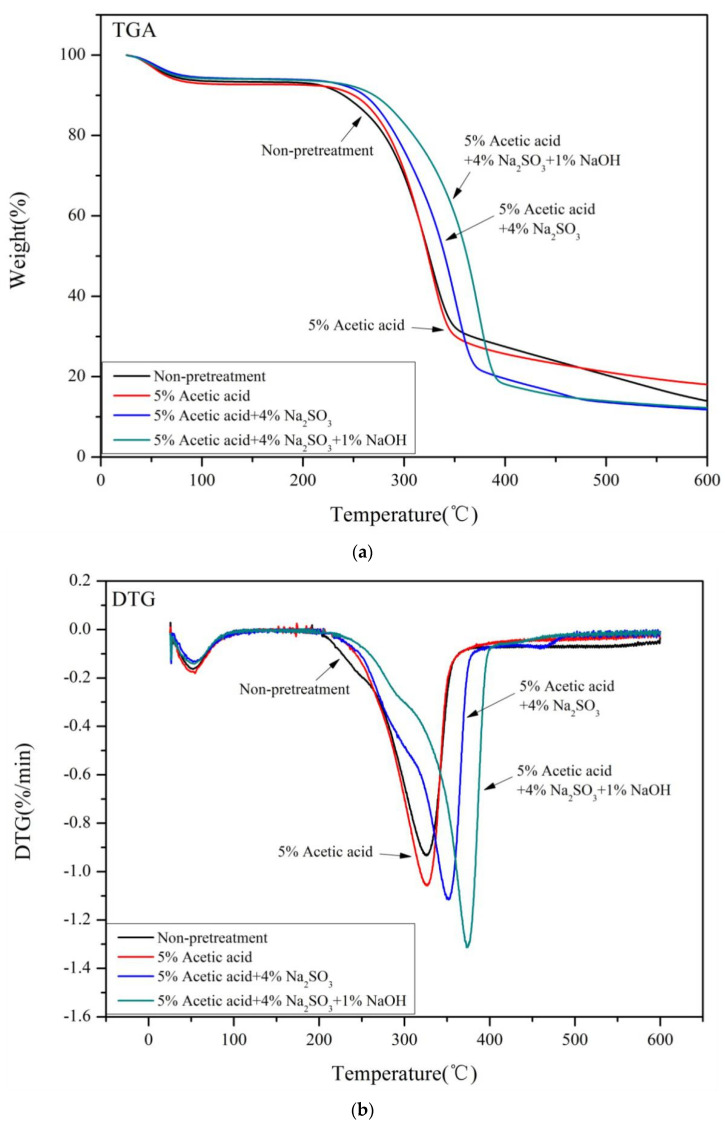
(**a**) TGA curves of samples under different pretreatment conditions. (**b**) DTG curves of samples under different pretreatment conditions.

The TGA curves of the four pretreated samples had some similarities, as shown in Figure 3. The pyrolysis of the samples was divided into four stages: the moisture removal stage (50–125 °C), the hemicellulose degradation stage (180–320 °C), the cellulose degradation stage (320–400 °C), and the lignin degradation stage (>360 °C) [35]. The weight loss between 0 and 150 °C was mainly due to the evaporation of water and other small molecules. The non-treated samples began to degrade at about 200 °C, while the pretreated samples began to degrade at a higher temperature (~225 °C), which became faster upon increasing the temperature (Figure 3a). Therefore, all pretreatments reduced the thermal stability of the Paulownia samples, mainly due to the full removal of lignin and the partial removal of hemicelluloses, as well as an increase in the cellulose content and crystallinity [41]. The weight loss at 350 °C was mainly caused by the degradation of lignin and cellulose. The thermal degradation rate at this stage was relatively slow, most likely due to the high thermal stability of lignin [42]. The degradation of lignin continued above 600 °C, with a residue of 14–20%. The lignin content in the samples pretreated with Na_2_SO_3_–NaOH was lower than that of the samples pretreated with only Na_2_SO_3_. The remaining residues were similar, possibly because of the different lignin structures.

Figure 3b shows the first differential curve (DTG) of the thermogravimetric curve. Most sample degradation occurred between 200 °C and 400 °C. The degradation temperature of hemicelluloses was 323 °C, while the degradation temperature of pure cellulose was 356 °C. The thermal degradation of lignin occurred slowly and over a wide temperature range, so the degradation was difficult to confirm. The DTG curves of the non-treated samples showed a broad peak at 200–400 °C. The maximum peaks of the pretreated samples shifted to higher temperatures compared with the non-treated samples. The maximum thermal degradation temperatures of cellulose in the samples pretreated with acetic acid, Na_2_SO_3_, and Na_2_SO_3_–NaOH were 326 °C, 352 °C, and 374 °C, respectively. The non-treated samples degraded at 324 °C, possibly due to their higher ash content, which may act as a catalyst during pyrolysis to accelerate the degradation of cellulose [43]. The maximum degradation rates of cellulose in all pretreated samples increased, possibly due to the higher cellulose crystallinity of the samples after pretreatment [44].

### 3.5. XPS Analysis

**Figure 4:**
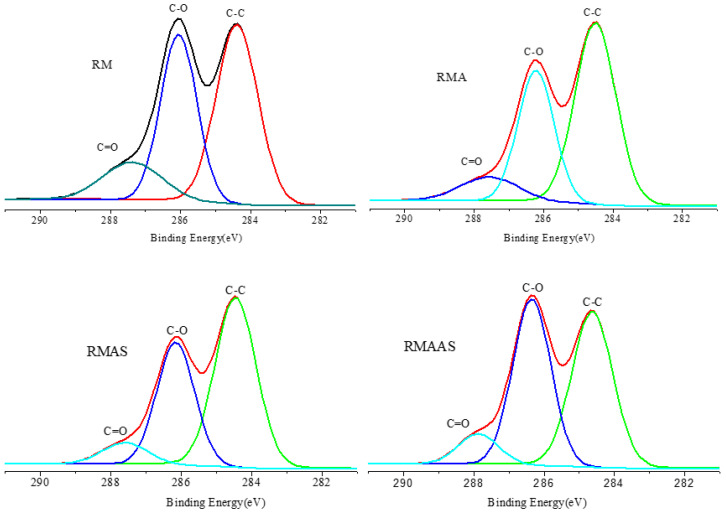
XPS spectra of samples after different pretreatment methods. No pretreatment (RM); 5% acetic acid (RMA); 5% acetic acid + 4% Na_2_SO_3_(RMAS); 5% acetic acid + 4% Na_2_SO_3_ + 1% NaOH(RMAAS).

Figure 4 shows the XPS spectra of the *Paulownia elongate* samples. The content of C-C bonds decreased and the content of C-O bonds increased after sodium sulfite pretreatment. This shows that alkaline sodium sulfite pretreatment improved the removal of lignin from the surface of samples.

**Figure 5:**
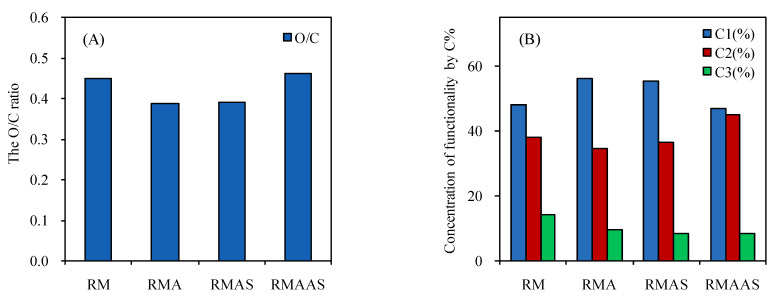
The O/C ratio (**A**) and concentration of functionality by percentage carbon (**B**) in the samples after different pretreatments. No pretreatment (RM); 5% acetic acid (RMA); 5% acetic acid + 4% Na_2_SO_3_ (RMAS); 5% acetic acid + 4% Na_2_SO_3_ + 1% NaOH (RMAAS). C-C/C-H(C1); (C-O)C2; (C=O)C3.

The oxygen-carbon ratio (O/C) of the samples was calculated based on the carbon-oxygen composition of the biomass surface layer (2–10 nm) (Figure 5). The O/C ratios of the main chemical groups were in the following order: cellulose and GT; hemicelluloses and GT; lignin and GT; extractives. The theoretical O/C ratios of cellulose, hemicelluloses, lignin, and extractives were 0.83, 0.33, and 0.09, respectively [45]. In this study, the O/C ratio of non-treated samples was 0.45, which decreased after acetic acid pretreatment to 0.387, which was close to the theoretical value of lignin. This may be due to the degradation of some xylan during acetic acid pretreatment and an increase in the lignin content. After Na_2_SO_3_ pretreatment, the O/C ratio increased to 0.39, which is still very close to the theoretical value of lignin. In general, the pretreatment processes removed a large number of extractive compounds. There was still a large amount of lignin on the surface layer after pretreatment with Na_2_SO_3_, but the total amount of lignin was greatly decreased (Table 1). This may be due to the redeposition of lignin during Na_2_SO_3_ pretreatment and washing. A high O/C ratio indicates a high cellulose and/or hemicelluloses content, and a low O/C ratio indicates a high lignin content [46]. Therefore, the increase in the O/C ratio indicated a decrease in the lignin content on the surface layer. After Na_2_SO_3_–NaOH pretreatment, the O/C ratio increased to 0.46, which indicated that the lignin content on the surface decreased; therefore, the synergistic pretreatment of Na_2_SO_3_ and NaOH promoted the removal of total lignin and surface lignin. As discussed in Section 3.1, the lignin and carbohydrate contents of the samples treated with alkaline sodium sulfite significantly decreased, indicating that it also benefitted from enzymatic hydrolysis.

The concentration of functional groups was analyzed based on the carbon content after different pretreatment conditions (Figure 5). The high-resolution carbon peaks were divided into four categories: C-C/C-H (C1), C-O (C2), C=O (C3), and COOH (C4) [35]. In the samples, only C1, C2, and C3 peaks were observed, which was similar to the C1 of corn stalk. The C1 peak was mainly composed of lignin and extractives. Compared with the non-treated samples, the C1 content in the samples treated with acetic acid was higher, probably due to the removal of xylan, which increased the lignin content on the surface. The C1 content was only slightly lower after sodium sulfite pretreatment, indicating that the surface layer was still covered with lignin, and some of the extractives may have been removed. These results were similar to those of previous studies.

The C1 content of the treated wood was much lower than that of the non-treated wood after Na_2_SO_3_ pretreatment, which confirmed the decrease in the lignin content on the surface. The addition of NaOH to this pretreatment method promoted the removal of lignin. It is commonly perceived that the C2 and C3 peaks are associated with polysaccharides, including cellulose and hemicelluloses. The content of C2 (surface carbohydrate) was significantly increased after Na_2_SO_3_–NaOH pretreatment.

### 3.6. Enzymatic Hydrolysis by Cellulase

#### 3.6.1. Effects of Cellulase Dosage on Hydrolysis Yields

**Figure 6:**
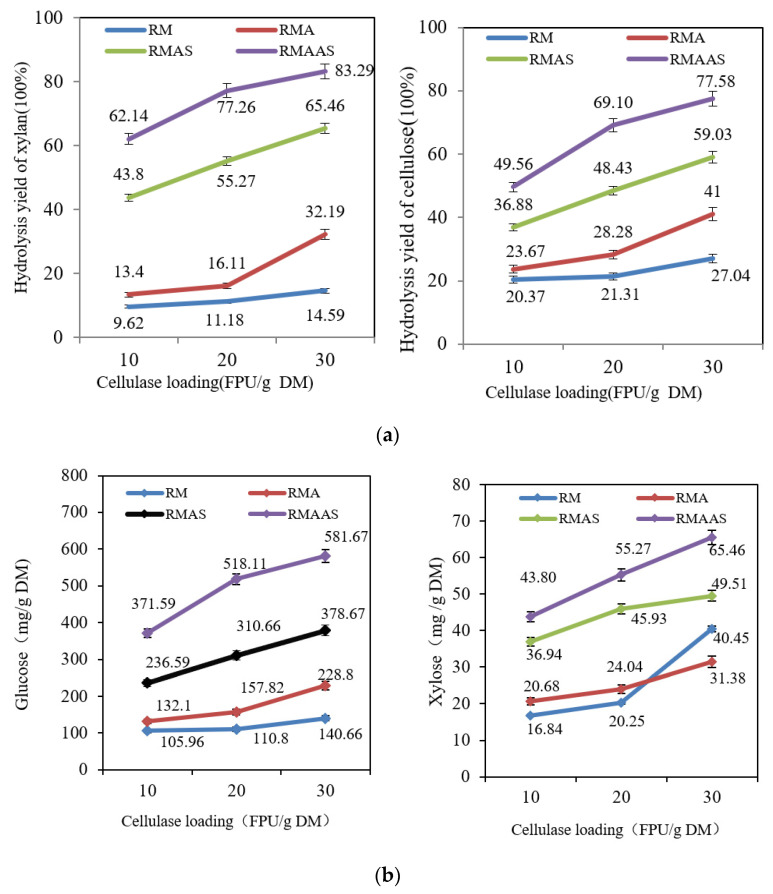
(**a**) Hydrolysis yields (%) of samples subjected to different pretreatment methods according to cellulase dosage. Cellic^®^CTec2 (10, 20, 30 FPU/g DM) at 50 °C and pH 5.0 for 72 h. (**b**) Hydrolysis yields (mg/g DM) in samples subjected to different pretreatment methods according to cellulase dosage. Cellic^®^CTec2 (10, 20, 30 FPU/g DM) at 50 °C and pH 5.0 for 72 h. RM: Raw material, no pretreatment. RMA: Raw material + 5% acetic acid. RMAS: Raw material + 5% acetic acid + 4% Na_2_SO_3_. RMAAS: Raw material + 5% acetic acid + 4% Na_2_SO_3_ + 1%NaOH.

Figure 6a shows that the hydrolysis yield under the tested conditions increased upon increasing the cellulase dose. For the non-treated samples, when the cellulase dose was increased from 10 FPU/g DM to 30 FPU/g DM, the glucose yield increased from 20% to 27%, and the xylose yield increased from 10% to 15%. Upon increasing the cellulase dosage, the hydrolysis yield was only slightly improved. The hydrolysis yield was improved after acetic acid pretreatment compared with the non-treated material: when the cellulase dose was 30 FPU per gram DM, the glucose yield increased by 14%. This may be due to the high-temperature acetic acid pretreatment, which degraded some hemicelluloses, which destroyed the complex structure between the three components [38]. This increased the specific adsorption between cellulase and cellulose and further improved the enzymatic hydrolysis yield.

After pretreatment with Na_2_SO_3_, the hydrolysis yield was significantly improved. When the cellulase dose was 10 FPU per gram DM, the glucose yield was 17% higher, and the xylose yield was 34% than the non-treated material. When the cellulase dose was increased to 30 FPU per gram DM, the glucose yield was 32% higher, and the xylose yield was 51% higher than the non-treated material. This was attributable to the good removal of lignin and lignin sulfonation by sodium sulfite [39]. After removing the lignin component, the crosslinked structure between the three elements was destroyed, and the structure of the substrate became loose and porous, which exposed more carbohydrate components that could be hydrolyzed by enzymes into monosaccharides [40].

The synergistic Na_2_SO_3_–NaOH treatment provided the best hydrolysis effect, and the yields of glucose and xylose were greatly improved. When the cellulase dose was 30 FPU per gram DM, the glucose yield reached 78%, and the xylose yield reached 83%. This was because alkaline sodium sulfite removed more lignin, providing more efficient lignin sulfonation which increased the accessibility of cellulose to cellulase and increased the hydrophilicity of lignin. The introduction of sodium hydroxide decreased the total lignin content and surface lignin content, thereby increasing the glucose yield.

Figure 6b shows that the hydrolysis yield (in mg/g DM) also increased upon increasing the cellulase dose. The synergistic alkaline sodium sulfite treatment worked the best. When the cellulase dosage was 10 FPU per gram DM, the glucose yield was 372 mg/g DM, and when the dosage was increased to 30 FPU per gram DM, the yield increased to 582 mg/g DM. Compared with the non-treated samples, the yield increased by 441 mg/g DM. In addition, as the cellulase dosage was increased, the xylose yield of the samples increased, but the total yield was small, only 65 mg/g DM. After acetic acid treatment, the xylose yield was slightly reduced, possibly due to the degradation of some xylan [40]. After Na_2_SO_4_ treatment, the xylose yield increased, but the total yield was only 44–65 mg/g DM. After synergistic treatment with alkaline sodium sulfite, the xylose yield was partially reduced.

#### 3.6.2. Effects of Hydrolysis Time on Hydrolysis Yields

**Figure 7:**
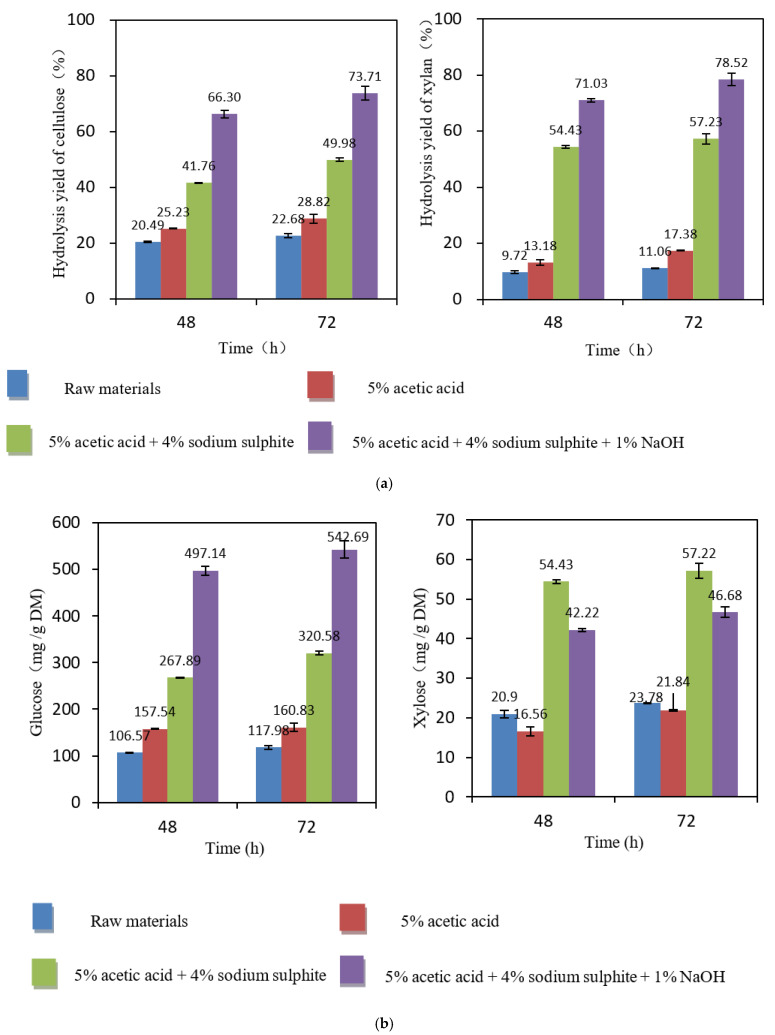
(**a**) Hydrolysis yields (%) after different pretreatments of samples according to the enzymatic hydrolysis time. Cellic^®^CTec2 (20 FPU/g DM) at 50 °C and pH 5.0 for 48 h and 72 h. (**b**) Hydrolysis yields (mg/g DM) after different pretreatments of the samples according to the enzymatic hydrolysis time. Cellic^®^CTec2 (20 FPU/g DM) at 50 °C and pH 5.0 for 48 h and 72 h.

Figure 7a shows that the hydrolysis yield slightly increased when the hydrolysis time was increased from 48 h to 72 h when the enzyme dosage was 20 FPU per gram DM. Prolonging the hydrolysis time had a similar effect on the hydrolysis of cellulose and xylan, both showing increased yields. After 48 h of hydrolysis of the non-treated samples, the glucose yield was 20%, and the xylose yield was 10%. After 72 h of hydrolysis, the hydrolysis yields increased slightly by about 2%. This may be due to the close connections between the three major chemical groups in the non-treated natural samples, where the cellulase did not easily contact the substrate [41].

After acetic acid pretreatment, the hydrolysis yield was only slightly higher, but after pretreatment with Na_2_SO_3_, the hydrolysis yield was significantly improved. When the hydrolysis time was 48 h, the yields of glucose and xylose were 42% and 54%, respectively. After prolonging the hydrolysis time to 72 h, the yields of glucose and xylose increased to 50% and 57%, which were 8% and 3% higher, respectively.

Compared with the non-treated material, the yields were significantly improved after synergistic Na_2_SO_3_–NaOH treatment. When the hydrolysis time was 48 h, the yields of glucose and xylose were 66% and 71%, respectively. After prolonging the hydrolysis time to 72 h, the yields of glucose and xylose increased to 74% and 79%.

Figure 7b shows that the hydrolysis yield also slightly increased when the hydrolysis time was increased from 48 h to 72 h at an enzyme dosage of 20 FPU per gram DM. The best yields and shortest hydrolysis times were obtained using the synergistic Na_2_SO_3_–NaOH treatment. The glucose yield was 497 mg/g DM after 48 h of hydrolysis. When the hydrolysis time was extended to 72 h, the glucose yield increased to 543 mg/g DM, which was higher than that of the non-treated samples (425 mg/g DM). The increase may be because the alkaline sodium sulfite removed more of the other chemical compounds (hemicelluloses, lignin, extractives), thereby promoting efficient adsorption between cellulase and cellulose. In addition, the xylose yield generally increased upon prolonging the hydrolysis time, but it decreased slightly after acetic acid treatment, possibly due to the degradation of some xylan. After Na_2_SO_3_ treatment, the xylose yield increased, but the total amount was only 54–57 mg/g DM. After the synergistic Na_2_SO_3_–NaOH treatment, the yield of xylose was partially decreased. Accordingly, the addition of NaOH and Na_2_SO_3_ greatly promoted the enzymatic hydrolysis and increased the glucose concentration in the hydrolysate. Their combined effects enhanced the hydrolyzability of Paulownia after alkaline sulfite pretreatment. This indicated that Na_2_SO_3_–NaOH pretreatment is an efficient method for producing fermentable sugars, which is in good agreement with the results of previous studies performed on various bio-based materials [42,43,44,45,46].

## 4. Conclusions

The synergistic treatment of *Paulownia elongate* with alkaline sodium sulfite (acetic acid, sodium sulfite, and sodium hydroxide) showed excellent enzymatic hydrolyzability. The sulfonation of lignin and delignification improved the enzymatic digestibility of the pretreated Paulownia samples. Pretreatment with acetic acid destroyed the crosslinked structure between the three major chemical compounds to some extent and also degraded some xylan components; however, it only slightly promoted hydrolysis. The combined treatment with acetic acid and sodium sulfite removed more lignin and exposed more cellulose, which increased the effective contact between cellulase and cellulose. The synergistic alkaline sodium sulfite treatment exhibited superior enzymatic hydrolysis characteristics, while also destroying the structure of the substrate.

## Figures and Tables

**Table 1:** Chemical composition of samples under different pretreatment conditions: average values and ranges of variation.

Pretreatment	Cellulose (%)	Xylan (%)	Lignin (%)
Non-pretreatment (RM)	46.81 ± 0.83 ^b^	18.92 ± 0.64 ^a^	31.98 ± 1.35 ^b^
5% acetic acid (RMA)	50.23 ± 1.27 ^b^	11.06 ± 0.26 ^a^	38.09 ± 1.28 ^a^
5% acetic acid + 4% Na_2_SO_3_ (RMAS)	57.73 ± 0.09 ^b^	8.80 ± 0.21 ^a^	34.15 ± 0.43 ^b^
5% acetic acid + 4% Na_2_SO_3_ + 1% NaOH (RMAAS)	67.48 ± 0.22 ^a^	5.23 ± 0.07 ^a^	26.38 ± 0.49 ^b^

Different letters in the same column indicate significant differences (*p* < 0.05).

## Data Availability

The data presented in this study are available on request from the corresponding authors.
